# “*Even Though the System Had Failed Him His Entire Life, We Were Failing Him Yet Again*”: How Clinical, Welfare, and Penal Medicine Interact to Drive Health Inequities and Medical Moral Injury

**DOI:** 10.3390/healthcare12131354

**Published:** 2024-07-07

**Authors:** Siddhi S. Ganesh, Kyle B. Joyner, Shamsher Samra, Ricky N. Bluthenthal, Todd W. Schneberk

**Affiliations:** 1 Department of Population and Public Health Sciences, Keck School of Medicine, University of Southern California, Los Angeles, CA 90032, USA; rbluthen@usc.edu; 2 Los Angeles General Medical Center, Department of Emergency Medicine, Keck School of Medicine, University of Southern California, Los Angeles, 1200 N State St, Los Angeles, CA 90033, USA; todd.schneberk@med.usc.edu (T.W.S.); Kyle.joyner@med.usc.edu (K.B.J.); 3 Department of Emergency Medicine, David Geffen School of Medicine, University of California, Los Angeles, CA 90095, USA

**Keywords:** substance use disorder, incarceration, medication for opioid use disorder, harm reduction, moral injury

## Abstract

Adam, a justice-involved young man, was brought into the emergency department at the county hospital in cardiogenic shock due to a recurring episode of injection-drug-use-related infective endocarditis (IDU-IE). Adam had initiated injection opioid use in prison. He was surgically treated for the previous episodes of IDU-IE but was unable to fully recover due to limitations in care within penal medicine. This case report explores the prison as a determinant of health, interactions between clinical, welfare, and penal medicine^,^ to produce and maintain health inequities, and structural drivers of physician moral injury through an interview with Adam and reflexive writings from emergency medicine physicians. This case demonstrates the need for three types of structural health interventions: (1) restorative justice, community-based reentry programs, and housing as welfare medicine, (2) increased harm reduction services across healthcare, especially penal medicine, and (3) equitable institutional protocols (contrary to ambiguous guidelines) to treat clinical conditions like IDU-IE that disproportionately impact structurally vulnerable patients.

## 1. Background 

Adam had a history of congenital heart disease. He was born with a bicuspid aortic valve, resulting in symptomatic aortic stenosis that was treated surgically in his childhood. Growing up with eight other siblings, when he was ten years old, his stepfather told him he was adopted. Looking back at this major turning point, Adam said the following:

“*I started acting out…As I got older, it just became a cycle of me getting in and out of jail. And then when I turned 18, I started going to the county jail. And then I started doing time in prison…I had never seen heroin until I went to prison. I caught hep-C in prison…Prison didn’t really teach me anything.*”

Upon leaving prison, Adam started experiencing opioid withdrawal symptoms and sought illicit market heroin to manage these symptoms. Soon after, he was diagnosed with his first episode of injection-drug-use-related infective endocarditis (IDU-IE) and was treated surgically. However, due to failures in medical care at different carceral facilities, he was not provided with medications to complete his outpatient antibiotic treatment course. Around this time, Adam was diagnosed with deep vein thrombosis (DVT) in his leg and a second episode of IDU-IE. An additional surgery was performed to treat the recurrence of IDU-IE.

Post-surgery and release from prison, Adam looked for substance use treatment programs but was unable to enroll due to delays in medical record transfer requests and program ineligibility due to his complex medical history. This was exacerbated by the insurance transition from his carceral care provision back to Medicaid [1] and the delay in enrollment after application. Adam reinitiated substance use, attributing it to this lack of programs and services. To stop using opioids, he even moved away from his neighborhood and searched for housing programs, to no avail. Eventually, he was arrested and returned to jail due to his drug use. There, he started to feel unwell again. 

Obtaining health care while incarcerated included barriers to accessing physicians, inconsistencies in communication between providers, and access to medication. He shared the following:

“*In the county jail they treat you like they don’t care. Everything is like, ‘Oh, drink some water you’ll be alright, drink some water you’ll be alright’. [About the process of seeing a physician] You fill out a form…Once you fill out the form, it’s 13–14 days just for you to see the doctor. And then… It feels kinda hopeless you know. To fill out a form because it’s like, ‘Oh I have this wrong with me, if I fill out a form, by the time I get there, I’ll probably already be OK.’ So that’s how a lot of people feel like. I might go look for someone who has the pills I need. I went looking for pills for a cold. Even the nurses will give you the medicine because they know it will take forever to see the doctor. It’s just such as struggle even just to get anything then and there.*”

Adam spent crucial time waiting for a cardiology referral assured to him by one provider and later found out from another provider that he never had a formal referral. In jail, he did not have access to specific analgesic and antibiotic medications prescribed to him at his latest hospital discharge. Adam visited the physician five times prior to being transported to the local hospital for his most recent episode of IDU-IE. The day he was brought into the hospital, he woke up with shortness of breath, collapsed to the ground, and yelled “man down” multiple times before he was brought to the hospital. 

In the hospital, Adam was admitted to the cardiology service for heart failure from a third episode of recurrent IDU-IE. His symptoms began with mild shortness of breath and rapidly progressed to cardiogenic shock with multiorgan failure. He was diagnosed with severely complicated endocarditis with a valvular abscess requiring corrective surgery. Adam was denied a third valve surgery due to his history of substance use and died soon after. 

Prior to his death, the emergency medicine (EM) physicians caring for Adam called other hospitals to attempt transfer and involved their supervising institution’s ethics committee. Limited by his incarcerated status, the physicians were only able to successfully advocate for increased visitation rights to see his mother and partner before his passing. Adam provided written consent to publish the contents of his interview and the physician’s submitting reflexive writings. In this interview, he said the following:

“*I just, I just want a chance. A good, solid chance.*”

## 2. Social Analysis Concepts 

The United States incarcerates more people than any other independent democracy, disproportionately disenfranchising people who are African American, Hispanic, and Indigenous [2]. For instance, while 14% of the United States population is African American, they make up 35% of the incarcerated population [3]. Hyper-incarceration refers to this disproportionate incarceration rate and is a form of structural violence [4,5,6]. Structural violence describes the ways in which large-scale forces (such as racism and immiseration) and inequitable public policies (i.e., restricting public support for low-income people, criminalizing housing insecurity) become embedded within institutions (such as medicine), causing harm to specific individuals and communities [4,5,6] Within medical institutions, Seim and DiMario identified three terrains, namely, clinical, welfare, and penal medicine that have focused their interventions on the governing poor [1]. In this case study, we explore these interactions via experiences shared by Adam and his emergency medicine clinicians. 

While the United States constitution does not guarantee healthcare, incarcerated people have a right to the community standard of healthcare [2]. However, prisons and jails serve as determinants of health due to various overlapping and intersecting factors. These include exposure to infectious disease, access to treatment, and a paucity of resources for societal reentry to name a few [7]. Adam reported first injecting heroin in prison and continuing to use opioids upon release. IDU-IE is an infection of the heart valve that requires surgery and parenteral antibiotics for 6–8 weeks. People who inject opioids can develop IDU-IE from bacteria entering the bloodstream due to lack of sterile needles or sterile injection supplies, reusing or sharing needles, being injected by others, and substance contamination from lack of safe supply [8,9,10]. Multi-level barriers to providing Medications for Opioid Use Disorder (MOUD) in criminal justice settings can exacerbate these existing risks [11] Current American Association for Thoracic Surgery (AATS) guidelines on the surgical treatment of infective endocarditis recommend that “normal indications for surgery [should be] applied to people who use drugs (PWUD), but management must include treatment of addiction” [12] However, in Adam’s case, he was unable to access MOUD both in criminal justice settings and upon reentry. Beyond that, this case elucidates how substance use treatment extends far beyond MOUD and includes wrap-around services like housing, re-entry, and employment programs. 

Emergency departments (EDs) are often referred to as the safety net of the United States healthcare system, as EDs provide medical care to any and all patients regardless of insurance status [1,13,14]. ED’s provide care to materially deprived communities [1] and, consequently, EM physicians often take on a bridging role as providers of social emergency medicine [13] This has a profound effect on those practicing in the ED. Concurrently, one metanalysis showed that EM physicians had the highest levels of burnout, as high as 40%, and were more vulnerable to burnout than physicians from other departments [15]. According to the United States Department of Veterans Affairs and the National Center for Post-traumatic Stress Disorder (PTSD), under traumatic, stressful, or unprecedented circumstances, individuals may perpetrate, witness, and/or fail to prevent the occurrence of events that go against moral beliefs or expectations they hold deeply [16]. Moral injury can take place among healthcare workers if and when they have to make difficult decisions when triaging life-and-death cases and/or under circumstances involving resource allocation or when they believe they or the system should have been able to save a patient’s life but were not able to do so [16]. 

Adam’s case emphasizes three main social concepts: (1) hyper-incarceration [4] and prison as a determinant of health; (2) interactions between clinical, welfare, and penal medicine [1] to produce and maintain health inequities; and (3) moral injury as a structural phenomenon [16]. 

### 2.1. Hyper-Incarceration and Prison Are Determinants of Health 

The adverse events in his childhood and subsequent punitive interventions [17,18] facilitated his first injection opioid use in prison. Below, Adam describes the impact of that initiating heroin use in prison and the lack of structural support for substance use disorder (SUD) treatment. In prison, he participated in a computer program for substance use disorders but noted that no other treatment was available. Regarding infective endocarditis, he shared the following:

“*Then I went to prison again and I found out I had caught…endocarditis. And I got the surgery for endocarditis the first time. And after that, I didn’t get a chance to recover the right way. I got sent back to jail. I didn’t get sent to a program. I didn’t get the right transitioning into the life I should have lived. I went straight to jail. And in jail I started doing drugs again…[I did not have a] support system to help me fully. [A support system that was not], ‘It’s my way or the high way’, [instead a support system that said], ‘Here let me help you take a step forward, here let me help you step forward and take a step forward.*’”

(Adam)

He described the medical treatment he received in prison as follows:

“*When you guys send forms with us of recommendations and [they state] ‘this person is supposed to take this medication, and this medicine and this medicine’, when it gets there [i.e., prison], everything gets disregarded, and it goes down the hill. it goes to the doctor and the doctor says…Like the last time when I got antibiotics, the doctor told [me], ‘Oh, I don’t really think you need this.*’”

(Adam)

### 2.2. Clinical, Penal, and Welfare Medicine Interact to Produce and Maintain Patient Health Inequities 

Adam’s recurrent episodes of IDU-IE were a result of incarceration, lack of access to MOUD, antibiotic treatment discontinuity in prison [19,20], and subsequent inability to recover fully from his previous surgeries. Below, Adam’s clinician reflects on the factors that led to a recurrence of endocarditis:

“*Unfortunately, because of a combination of a lack of treatment follow-through, access to healthcare, and drug abuse programs in prison, Adam had a relapse of his endocarditis.*”

(One of Adam’s physicians)

Adam identified that his social network and neighborhood shaped his opioid use and identified the services that would have supported treatment. He said the following:

“*My drug addiction was just hard because [of] the people I hang out with, I go back to the same shit you know. That’s why I need a program to help me get into housing because when I don’t have nowhere to go, I go the hood.*”

He moved into to his sister’s home in an attempt “*to get away from the environment*”. When asked what prompted his return, he said the following:

“*I just didn’t have no money. So, I went back to where I knew I could get money, you know… It was the addiction and the lack of support that I had, I had my sister, but I didn’t have nobody else. So, I didn’t have anywhere to go, so like fuck it I’ll just go back home. I know that there’s programs where they take you and they build you and build you until you get home and they provide you with housing, provide with you like a job. Like if I was at my sisters and I had a job, I think I would be able to make it. Like, Suboxone clinics I think.*”

(Adam)

Adam attempted to get into a program but did not meet the eligibility criteria:

“*I went through the [recovery] and I tried to go check into a [substance use] program, and I needed to find the right program because that program wouldn’t take me due to my medical issues.*”

(Adam)

Aspects of Adam’s case elucidate how institutionalized deservingness [6] in clinical medicine operates via treatment futility and resource rationing [9] against disenfranchised and materially deprived patients [21,22] in this case, resulting in the determination of poor surgical candidacy [23].

“*...Our surgeon colleagues felt like they could not justifiably offer care to this poor individual due to it being too high risk, a short-term solution, and unfair from a resource standpoint to perform a third surgery.*”

(One of Adam’s physicians)

Here, Adam’s physician describes how his structural vulnerabilities (incarcerated status and complex history of recurring IDU-IE) further limited their ability to care for him:

“*We made phone calls to other hospitals but were limited by his incarcerated status and complex history.*”

(One of Adam’s physicians)

### 2.3. Medical Moral Injury Is Structurally Driven 

In medicine, moral injury via commission occurs when a clinician acts against their beliefs, whereas moral injury via omission takes place when a clinician is unable to act on their deeply held beliefs [16] In times of overlapping public health crises, such the intersection of the opioid mortality crisis, dissolving social safety net, inaccessible wrap-around support, and hyper-incarceration in Adam’s case, clinicians may witness what they believe to be unjustifiable/unfair acts or policies that may lead to a feeling of betrayal from individuals, systems, and institutions of power [16]. The reflexive ethnographic writings by Adam’s EM clinicians on this case demonstrate how an institutional failure [19] to account for the needs of structurally vulnerable patients [5] displaces that burden onto individual-level clinicians who feel restricted in their ability to provide care:

“*When his life intersected with ours in healthcare, I feel like he had been failed so many times by society and we did not have the ability to help him.*”

(One of Adam’s physicians)

“*We had to tell Adam that there was nothing we could do for him. We had to tell him, that even though the system had failed him his entire life, we were failing him yet again, and this time our failures were going to cost him his life.*”

(One of Adam’s physicians)

Such cases are stressful and procedurally ambiguous, and clinicians are constrained by structural vulnerabilities such as incarceration status that do not have clear and equitable guidelines for care. Altogether, these may result in moral injury [16,22].

“*The case has stuck with me more than any other as a physician because I have never been in the situation of telling a [young patient] that they will die of an illness where we typically do have interventions to offer.*”

(One of Adam’s physicians)

“*I have question[ed] if I felt like the principle of justice was not honored in his final days. He had self-admittedly made serious mistakes and poor life choices that resulted in him being in this situation. But he also expressed a desire to change, to seek treatment, and to try to turn his life around. He also was born with a congenital condition that predisposed him to this disease, suffered from adverse childhood experiences, and had been in the cycle of incarceration and substance use—these have all been studied in medical and psychological literature to be outside of an individual’s control.*”

(One of Adam’s physicians)

## 3. Discussion

Adam’s case emphasizes how structural failures such as hyper-incarceration and punitive justice in the United States result in outcomes that are displaced onto institutions, such as hospitals and medical systems [1,4]. Here, they remain unaddressed due to a lack of bureaucratic protocols and guidelines outlined for incarcerated patients, resource rationing and deservingness, and because of conditions arising from the structural failures themselves. For instance, Adam was declared a poor candidate for surgery due to failures of care in the penal healthcare system, i.e., barriers to medication adherence, MOUD access, and not being able to see a doctor on time [1,8,9,11]. Within medicine, the downstream impacts of institutional failures are displaced onto frontline workers, including physicians, nurses, social workers, social services workers, and program coordinators, who attempt to navigate medical systems alongside structurally vulnerable patients [1,5]. At this point, frontline workers face barriers to access, care, and treatment from policy failures that are far beyond their clinical scope. In Adam’s case, clinicians faced barriers resulting from structural vulnerabilities like incarceration and housing insecurity that can increase propensity for moral injuries [1,16,21]. These moral injuries occur due to witnessing, acting, or being unable to act in ways that align with a provider’s core beliefs about how clinical care should be practiced and delivered [16]. This case underscores how structurally driven failures are embedded into institutions via deservingness and futility, resulting in morally injurious events for treating clinicians [9,24,25]

## 4. Implications

Adam’s case demonstrates the need for three types of structural health interventions: 

1. Restorative justice, community-based reentry programs, and housing as welfare medicine: Punitive justice and hyper-incarceration in the United States is a racist and anti-immigrant complex [26]. Restorative justice [26] and public investment in community building services [18] that may have prevented Adam from being incarcerated are necessary structural and preventive measures.

2. Increased harm reduction services across healthcare especially, penal medicine: Adam repeatedly mentioned the lack of support he received upon release from prison, especially for his substance use. This only emphasizes the need for harm-reduction-based care during the re-entry period, which is a particularly challenging time and a major missed opportunity for intervention.

3. Equitable institutional protocols (contrary to ambiguous guidelines) to treat clinical conditions like IDU-IE that disproportionately impact structurally vulnerable patients: Developing these protocols and guidelines with structurally vulnerable patient populations at the center is imperative for both patients and providers. To provide patient-centered care, protocols must include lived experience. For providers, equitable guidelines may reduce the risk of moral injury. 

## 5. Conclusions

Adam and his physicians underscore how reframing care to focus on patient-centered approaches is a multidimensional approach to mitigating clinician moral injury. A patient-centered approach to medicine that develops interventions for structurally vulnerable patients via clinical, welfare, and penal medicine is a key step towards addressing structural moral injury. Some of the intersecting effects of structural drivers like housing, incarceration, and a lack of programmatic support with ambiguity surrounding treatment guidelines for patients like Adam can be addressed on an institutional level. Consequently, targeted structural and institutional-level approaches that prioritize the wellbeing of Adam and other patients in similar circumstances may prevent clinicians from experiencing barriers to care provision. These can range from MOUD access to family visitation for incarcerated patients. In hospital settings, these would necessitate developing protocols to streamline care of incarcerated patients from the standpoint of what is medically necessitated and medically indicated, as well as identifying key barriers to care for incarcerated patients and leveraging institutional resources to address them.

## Abbreviations

DVT	Deep vein thrombosis
ED	Emergency department
EM	Emergency medicine
IDU-IE	Injection-drug-use-related infective endocarditis
MOUD	Medications for opioid use disorder
PTSD	Post-traumatic stress disorder

## References

[1] Seim J., DiMario A. (2023). City of Gauze: Medicine and the Governance of Urban Poverty. Soc. Probl..

[2] Alsan M., Yang C.S., Jolin J.R., Tu L., Rich J.D. (2023). Health care in U.S. correctional facilities—A limited and threatened constitutional right. N. Engl. J. Med..

[3] Sawyer W., Wagner P., Mass Incarceration: The Whole Pie 2024 Prison Policy Initiative. https://www.prisonpolicy.org/reports/pie2024.html.

[4] Karandinos G., Bourgois P. (2019). The structural violence of hyperincarceration—A 44-year-old man with back pain. N. Engl. J. Med..

[5] Bourgois P., Holmes S.M., Sue K., Quesada J. (2017). Structural vulnerability: Operationalizing the concept to address health disparities in clinical care. Acad. Med..

[6] Farmer P., Nizeye B., Stulac S., Keshavjee S., Bourgois P., Holmes S.M., Sue K., Quesada J. (2016). Structural violence and clinical medicine. Understanding and Applying Medical Anthropology.

[7] Klein D.E., Lima J.M. (2021). The prison industrial complex as a commercial determinant of health. Am. J. Public Health.

[8] Tiako M.J.N., Hong S.B., Bin Mahmood S.U.M., Mori M., Mangi A., Yun J., Juthani-Mehta M., Geirsson A. (2020). Inconsistent addiction treatment for patients undergoing cardiac surgery for injection drug use-associated infective endocarditis. J. Addict. Med..

[9] Hayden M., Moore A. (2020). Attitudes and Approaches Towards Repeat Valve Surgery in Recurrent Injection Drug Use-associated Infective Endocarditis: A Qualitative Study. J. Addict. Med..

[10] Schranz A., Barocas J.A. (2020). Infective endocarditis in persons who use drugs: Epidemiology, current management, and emerging treatments. Infect. Dis. Clin..

[11] Grella C.E., Ostile E., Scott C.K., Dennis M., Carnavale J. (2020). A scoping review of barriers and facilitators to implementation of medications for treatment of opioid use disorder within the criminal justice system. Int. J. Drug Policy.

[12] Pettersson G.B., Hussain S.T. (2019). Current AATS guidelines on surgical treatment of infective endocarditis. Ann. Cardiothorac. Surg..

[13] Salhi R., Macias-Konstantopoulos W.L., Ryus C.R. (2023). From the Editors—Future Directions to Strengthen the Emergency Department Safety Net. West. J. Emerg. Med..

[14] Centers for Medicare and Medicaid Services (2023). Emergency Medical Treatment & Labor Act (EMTALA). https://www.cms.gov/medicare/regulations-guidance/legislation/emergency-medical-treatment-labor-act.

[15] Zhang Q., Mu M.C., He Y., Cai Z.L., Li Z.C. (2020). Burnout in emergency medicine physicians: A meta-analysis and systematic review. Medicine.

[16] Norman S.B., Maguen S. (2020). Va.gov: Veterans Affairs. Moral Injury. https://www.ptsd.va.gov/professional/treat/cooccurring/moral_injury.asp.

[17] Stensrud R.H., Gilbride D.D., Bruinekool R.M. (2019). The childhood to prison pipeline: Early childhood trauma as reported by a prison population. Rehabil. Couns. Bull..

[18] Henry B.F. (2020). Adverse experiences, mental health, and substance use disorders as social determinants of incarceration. J. Community Psychol..

[19] Gonzalez J.M.R., Connell N.M. (2014). Mental health of prisoners: Identifying barriers to mental health treatment and medication continuity. Am. J. Public Health.

[20] Curran J., Saloner B., Winkelman T.N., Alexander G.C. (2023). Estimated use of prescription medications among individuals incarcerated in jails and state prisons in the US. JAMA Health Forum.

[21] DiMario A. (2022). To Punish, Parent, or Palliate: Governing Urban Poverty through Institutional Failure. Am. Sociol. Rev..

[22] Hansen H., Bourgois P., Drucker E. (2014). Pathologizing poverty: New forms of diagnosis, disability, and structural stigma under welfare reform. Soc. Sci. med..

[23] Muncan B., Kim E.K., Amabile A., Weimer M.B., Nguemeni Tiako M.J., Vallabhajosyula P., Kalogeropoulos A.P., Geirsson A. (2022). Cardiac surgeons’ perspectives and practices regarding people who use drugs: A scoping review. J. Card. Surg..

[24] Griffin B.J., Purcell N., Burkman K., Litz B.T., Bryan C.J., Schmitz M., Villierme C., Walsh J., Maguen S. (2019). Moral injury: An integrative review. J. Trauma. Stress.

[25] Dean W., Talbot S.G. (2020). Hard hits of distress. J. Pediatr. Rehabil. Med..

[26] González T. (2023). Restorative Justice Diversion as a Structural Health Intervention in the Criminal Legal System. J. Crim. Law Criminol..

